# Supplementary material for: Polyketide synthase gene clusters in the ericoid mycorrhizal fungus Oidiodendron maius: genome-wide prediction, expression profiling, and functional characterization of knockout mutants
Source: Front Plant Sci. 2026 Sep 16;17:1924430. doi: 10.3389/fpls.2026.1924430 (PMC13623903; doi:10.3389/fpls.2026.1924430)
Supplement: Supplementary file 1 [file SupplementaryFile1.zip › Supplementary Materials/Supplementary Folder 1.zip!/Oidma1_AssemblyScaffolds_Repeatmasked/knownclusterblast/cluster17/jgi.p_Oidma1_40858_mibig_hits.html]

| MIBiG Protein | Description | MIBiG Cluster | MiBiG Product | % ID | % Coverage | BLAST Score | E-value |
| --- | --- | --- | --- | --- | --- | --- | --- |
| ABJ90146.1 | MoeA4 | BGC0000805 | Moenomycin\_biosynthetic\_gene\_cluster | 30 | 23.1655548817 | 135 | 4.8e-31 |
| ADC79613.1 | BafX | BGC0000028 | Bafilomycin\_biosynthetic\_gene\_cluster | 25 | 31.2916919345 | 134 | 8.2e-31 |
| AAN74813.2 | Fum10p | BGC0000062 | Fumonisin\_biosynthetic\_gene\_cluster | 25 | 30.6246209824 | 129 | 2.6e-29 |
| ACB12556.1 | Fum10 | BGC0000063 | Fumonisin\_biosynthetic\_gene\_cluster | 25 | 33.1109763493 | 128 | 7.7e-29 |
| AIL50189.1 | putative\_5-aminolevulinate\_CoA-ligase | BGC0000213 | Colabomycin\_biosynthetic\_gene\_cluster | 26 | 30.3820497271 | 128 | 1e-28 |
| ADI58647.1 | AsuD3 | BGC0000187 | Asukamycin\_biosynthetic\_gene\_cluster | 29 | 24.6816252274 | 126 | 2.2e-28 |
| AGY30675.1 | Ann3 | BGC0001298 | 4-Z-annimycin\_biosynthetic\_gene\_cluster | 28 | 28.5627653123 | 121 | 1.2e-26 |
| ABP55493.1 | thioester\_reductase\_domain | BGC0001006 | Lymphostin\_biosynthetic\_gene\_cluster | 31 | 24.4390539721 | 113 | 3.3e-24 |
| ACR50792.1 | putative\_acyl-CoA\_synthase | BGC0000163 | Tetronasin\_biosynthetic\_gene\_cluster | 28 | 21.8314129776 | 111 | 9.7e-24 |
| AKD43499.1 | Acyl-CoA\_ligase | BGC0001409 | Dutomycin\_biosynthetic\_gene\_cluster | 29 | 20.7398423287 | 110 | 1.7e-23 |
| ACN64850.1 | PokL | BGC0001061 | Polyketomycin\_biosynthetic\_gene\_cluster | 29 | 20.7398423287 | 101 | 1e-20 |
| CAQ52626.1 | type\_I\_polyketide\_synthase,\_loading\_module\_and\_modules\_1-3 | BGC0001066 | Kendomycin\_biosynthetic\_gene\_cluster | 28 | 21.7707701637 | 97 | 1.4e-19 |
| ACO31297.1 | PtmA3 | BGC0001140 | Platensimycin\_/\_platencin\_biosynthetic\_gene\_cluster | 26 | 21.2856276531 | 95 | 5.5e-19 |
| BAO66547.1 | cyclohexanecarboxylate-CoA\_ligase | BGC0000042 | Cremimycin\_biosynthetic\_gene\_cluster | 26 | 22.8623408126 | 93 | 2.1e-18 |
| ADD82992.1 | PtnA3 | BGC0001156 | Platencin\_biosynthetic\_gene\_cluster | 28 | 21.1036992116 | 92 | 4.7e-18 |
| AFV52184.1 | acyl-CoA\_synthetase/P450\_monooxygenase | BGC0000081 | Kedarcidin\_biosynthetic\_gene\_cluster | 28 | 21.7707701637 | 91 | 1e-17 |
| ABF88003.1 | putative\_long-chain-fatty-acid\_CoA\_ligase | BGC0000871 | VEPE\_/\_AEPE\_/\_TG-1\_biosynthetic\_gene\_cluster | 24 | 21.5281989084 | 89 | 5.2e-17 |
| CAE02600.1 | putative\_acyl-coA\_ligase | BGC0000024 | Aureothin\_biosynthetic\_gene\_cluster | 25 | 29.0479078229 | 87 | 2e-16 |
| CAI94705.1 | putative\_acid\_AMP\_ligase | BGC0000141 | Rubradirin\_biosynthetic\_gene\_cluster | 26 | 20.8004851425 | 85 | 7.4e-16 |
| CAO85890.1 | aryl-coA\_ligase | BGC0000110 | Neoaureothin\_biosynthetic\_gene\_cluster | 24 | 27.289266222 | 83 | 2.2e-15 |
| AAO07759.1 | 2,3-dihydroxybenzoate-AMP\_ligase | BGC0000460 | Vulnibactin\_biosynthetic\_gene\_cluster | 24 | 22.4378411158 | 83 | 2.8e-15 |
| CBK62739.1 |  | BGC0001115 | Thiomarinol\_biosynthetic\_gene\_cluster | 24 | 22.8623408126 | 82 | 4.8e-15 |
| CAI94686.1 | hypothetical\_protein | BGC0000141 | Rubradirin\_biosynthetic\_gene\_cluster | 27 | 21.2249848393 | 68 | 7.2e-11 |
| AFK57212.1 | DidA | BGC0000985 | Didemnin\_biosynthetic\_gene\_cluster | 24 | 26.0764099454 | 67 | 1.2e-10 |
| ADI58645.1 | AsuD1 | BGC0000187 | Asukamycin\_biosynthetic\_gene\_cluster | 23 | 21.1036992116 | 66 | 2.7e-10 |
| AIL50191.1 | putative\_amide\_synthetase | BGC0000213 | Colabomycin\_biosynthetic\_gene\_cluster | 26 | 21.3462704669 | 59 | 3.3e-08 |
| CAQ43084.1 | non\_ribosomal\_polypeptide\_synthetase | BGC0000970 | Chondrochloren\_biosynthetic\_gene\_cluster | 23 | 25.6519102486 | 53 | 2.4e-06 |
